# Excluded versus included patients in a randomized controlled trial of infections caused by carbapenem-resistant Gram-negative bacteria: relevance to external validity

**DOI:** 10.1186/s12879-021-05995-y

**Published:** 2021-03-31

**Authors:** Vered Daitch, Mical Paul, George L. Daikos, Emanuele Durante-Mangoni, Dafna Yahav, Yehuda Carmeli, Yael Dishon Benattar, Anna Skiada, Roberto Andini, Noa Eliakim-Raz, Amir Nutman, Oren Zusman, Anastasia Antoniadou, Giusi Cavezza, Amos Adler, Yaakov Dickstein, Ioannis Pavleas, Rosa Zampino, Roni Bitterman, Hiba Zayyad, Fidi Koppel, Yael Zak-Doron, Inbar Levi, Tanya Babich, Adi Turjeman, Haim Ben-Zvi, Lena E. Friberg, Johan W. Mouton, Ursula Theuretzbacher, Leonard Leibovici

**Affiliations:** 1 Sackler Faculty of Medicine, Tel Aviv University, Ramat-Aviv, Tel Aviv, Israel; 2 Department of Medicine E, Rabin Medical Center, Beilinson Hospital, Jebotinski 39, Petah Tikva, Israel; 3 Institute of Infectious Diseases, Rambam Health Care Campus, Haifa, Israel; 4 The Ruth and Bruce Rappaport Faculty of Medicine, Technion – Israel Institute of Technology, Haifa, Israel; 5 First Department of Medicine, Laikon General Hospital, Athens, Greece; 6 National and Kapodistrian University of Athens, Athens, Greece; 7 Internal Medicine, University of Campania ‘L Vanvitelli’, and AORN dei Colli-Monaldi Hospital, Naples, Italy; 8 Unit of Infectious Diseases, Rabin Medical Center, Beilinson Hospital, Petah Tikva, Israel; 9 Division of Epidemiology and Preventive Medicine, Tel Aviv Sourasky Medical Centre, Tel Aviv, Israel; 10 National Institute for Antibiotic Resistance and Infection Control, Ministry of Health, Tel Aviv, Israel; 11 Cheryl Spencer Department of Nursing, University of Haifa, Haifa, Israel; 12 Fourth Department of Medicine, Attikon University General Hospital, Athens, Greece; 13 Microbiology Laboratory, Tel-Aviv Sourasky Medical Center, Tel-Aviv, Israel; 14 Intensive Care Unit, Laikon General Hospital, Athens, Greece; 15 Clinical Microbiology Laboratory, Rabin Medical Center, Beilinson Hospital, Petah Tikva, Israel; 16 Department of Pharmaceutical Biosciences, Uppsala University, Uppsala, Sweden; 17 Department of Medical Microbiology and Infectious Diseases, Erasmus MC, Rotterdam, The Netherlands; 18 Center for Anti-Infective Agents, Vienna, Austria

**Keywords:** Population external validity, Antimicrobial resistance, Antibiotic treatment

## Abstract

**Background:**

Population external validity is the extent to which an experimental study results can be generalized from a specific sample to a defined population. In order to apply the results of a study, we should be able to assess its population external validity. We performed an investigator-initiated randomized controlled trial (RCT) (AIDA study), which compared colistin-meropenem combination therapy to colistin monotherapy in the treatment of patients infected with carbapenem-resistant Gram-negative bacteria. In order to examine the study’s population external validity and to substantiate the use of AIDA study results in clinical practice, we performed a concomitant observational trial.

**Methods:**

The study was conducted between October 1st, 2013 and January 31st, 2017 (during the RCTs recruitment period) in Greece, Israel and Italy. Patients included in the observational arm of the study have fulfilled clinical and microbiological inclusion criteria but were excluded from the RCT due to receipt of colistin for > 96 h, refusal to participate, or prior inclusion in the RCT. Non-randomized cases were compared to randomized patients. The primary outcome was clinical failure at 14 days of infection onset.

**Results:**

Analysis included 701 patients. Patients were infected mainly with *Acinetobacter baumannii* [78.2% (548/701)]. The most common reason for exclusion was refusal to participate [62% (183/295)]. Non-randomized and randomized patients were similar in most of the demographic and background parameters, though randomized patients showed minor differences towards a more severe infection. Combination therapy was less common in non-randomized patients [31.9% (53/166) vs. 51.2% (208/406), *p* = 0.000]. Randomized patients received longer treatment of colistin [13 days (IQR 10–16) vs. 8.5 days (IQR 0–15), *p* = 0.000]. Univariate analysis showed that non-randomized patients were more inclined to clinical failure on day 14 from infection onset [82% (242/295) vs. 75.5% (307/406), *p* = 0.042]. After adjusting for other variables, non-inclusion was not an independent risk factor for clinical failure at day 14.

**Conclusion:**

The similarity between the observational arm and RCT patients has strengthened our confidence in the population external validity of the AIDA trial. Adding an observational arm to intervention studies can help increase the population external validity and improve implementation of study results in clinical practice.

**Trial registration:**

The trial was registered with ClinicalTrials.gov, number NCT01732250 on November 22, 2012.

## Background

Randomized controlled trials (RCTs) are the gold standard for guidelines and evidence-based medicine. Internal validity of an RCT reflects the strengths to support a clinical decision based on study results and the extent to which the results are influenced by bias [1]. Adequate randomization, allocation concealment, blinding, non-selective reporting of outcomes and intention-to-treat analysis, have been identified as important factors in study design to minimize bias in RCTs and increase internal validity [1, 2]. External validity is defined as the extent and manner in which the results of an experimental study can be generalized to different subjects and settings. It has two components: population validity, the extent to which the results can be generalized from the specific sample to a defined population, and ecological validity, the extent to which the results can be generalized from the set of environmental conditions created by the researcher to other environmental conditions/settings [3].

The population external validity of RCTs relies firstly on the inclusion and exclusion criteria. Secondly, it relies on the population of patients actually recruited. Inclusion and exclusion criteria should be defined precisely, clearly and unambiguously [2]. Studies have shown that patients recruited into RCTs were sometimes different from those who were eligible but not recruited in terms of age, gender, educational status, socioeconomic status, place of residence, ability to provide informed consent and severity of disease. Patients that could not provide informed consent, and thus were not included, had more severe disease and their outcome was often worse compared to patients included in trials [4–6]. The problem of external validity is particularly relevant to registration trials, which typically specify numerous exclusion criteria. In order to apply a study’s results, one should be able to assess its population external validity; however, few studies to date have done so [7–12].

We performed an investigator-initiated, multicenter, open-label, parallel group, randomized controlled trial (AIDA study), which compared colistin-meropenem combination therapy to colistin monotherapy in the treatment of patients infected with carbapenem-resistant Gram-negative bacteria (CR GNB). The RCT differed from typical registration trials in its design, particularly in its broad eligibility criteria and in its limited exclusion criteria that were meant to reflect “real life patients”. The design, methods, and results have been previously published [13, 14]. In order to examine the study’s population external validity and to substantiate the use of AIDA study results in clinical practice, we performed a concomitant observational trial that compared the characteristics and outcomes of randomized (included) and non-randomized (excluded) AIDA study patients.

## Methods

### Study design and participants

We compared patients randomized in the trial (interventional arm) to those fulfilling clinical and microbiological inclusion criteria who were not randomized due to exclusion from the trial (observational arm).

The study was conducted between October 1st, 2013 and January 31st, 2017 (during the RCT recruitment period) in Laikon and Attikon Hospitals in Athens, Greece; Tel Aviv Sourasky Medical Center (Tel Aviv), Rabin Medical Center, Beilinson Hospital (Petah-Tikva) and Rambam Health Care Center (Haifa), Israel; and Monaldi Hospital, Naples, Italy.

Study population included adults (18+) with severe infections (requiring hospitalization or hospital acquired), caused by CR GNB that are susceptible to colistin, aminoglycosides, sulbactam, tetracyclines, tigecycline, and co-trimoxazole. Infections included bacteraemia, definite ventilator associated or hospital-acquired pneumonia, probable ventilator-associated pneumonia, and urosepsis. Polymicrobial infections comprising carbapenem-susceptible GNB were excluded from the RCT and from the observational arm.

Treatment in the interventional arm included intravenous colistin or colistin combined with meropenem. Colistin was administered as a 9-million unit (MIU) loading dose, followed by 4.5-MIU maintenance doses every 12 h, adjusted for renal function in patients with creatinine clearance of less than 50 mL/min. Meropenem was given as a 2 g extended-infusion (3 h) every 8 h, adjusted for renal function.

Patients excluded from the RCT for one or more reasons, but otherwise fulfilling clinical and microbiological inclusion criteria were included in the observational arm: refusal to participate; previous colistin treatment for more than 96 h at eligibility assessment; and prior inclusion in the RCT. Treatment in the observational arm was based on the attending physicians’ decisions.

### Outcomes

The primary outcome was clinical failure at 14 days after the first positive culture was obtained. The outcome was a composite of: patient deceased, systolic blood pressure < 90 mmHg or the need for vasopressor support, no stability or improvement in Sequential Organ Failure Assessment (SOFA) score, and for patients with bacteremia due to growth of the initial isolate in blood cultures taken on day 14. Secondary outcomes collected for this study were mortality at 14 and 28 days.

We also compared demographic data, background conditions, source of infection, devices present at infection onset, infection characteristics, and antibiotic treatment.

### Ethics

Both RCT and observational study were approved by local ethics committee in each site. Data on excluded patients (observational arm) were collected through electronic records. Informed consent was obtained for all RCT participants (interventional arm). In Israel, the RCT was approved as ‘emergency research’; patients who were not able to provide informed consent and did not have a legal guardian were included by the consent of an approved independent physician (providing direct patient care but not participating in the trial) and a family member. In Italy and Greece, a relative was an acceptable surrogate for patients that were unable to provide informed consent. In both cases, if the patient has improved, he was asked to provide an informed consent for participation. In the case of refusal, the patient was removed from the trial.

### Statistical analysis

Analyses were performed using the Statistical Package for the Social Sciences 25 (SPSS Inc.). Categorical data were compared using the chi-square test. A Kolmogorov-Smirnov test was carried out in order to determine whether the distributions of continuous variables were normal. Continuous variables were analyzed using t-test or Mann-Whitney-U test as appropriate. To examine risk factors for clinical failure on day 14 focusing on exclusion from the RCT, we performed a multivariable logistic regression. For the selection of our final model, we used Akaike’s Information Criterion. Nine models were tested to find the best fit. Different sets of significant variables (*p* < 0.1) were entered in consideration of clinical relevance. Interactions between exclusion from the RCT and other variables were not tested due to lack of clinical reasoning.”

## Results

Analysis was performed on 701 patients, including 295 non-randomized patients in the observational arm and 406 RCT patients. Patients were infected mainly with *Acinetobacter baumannii* [78.2% (548/701)].

The most common reason for not including suitable patients in the RCT was refusal to participate [62% (183/295)]. 20.7% (62/295) of patients were excluded due to treatment with colistin for more than 96 h, and 16.9% (50/295) were excluded for prior inclusion in the RCT.

### Patients’ characteristics

Non-randomized and RCT patients were similar in most of the demographic and background parameters. There were more patients with dementia in the RCT [10.7% (49/406) vs. 5.8% (17/295), *p* = 0.050]. Hematological malignancies were more common in non- randomized patients [8.5% (25/295) vs. 3.4% (14/406), *p* = 0.004]. At infection onset, RCT patients had more arterial lines [37.2% (151/406) vs. 25.8% (76/295), *p* = 0.001] central venous catheters [55.4% (225/406) vs. 40.3% (119/295), *p* = 0.000] and urinary catheters [87.2% (354/406) vs. 77.3% (228/295), *p* = 0.001] than non-randomized patients (Table 1).

**Table 1:** Patients’ characteristics

	Excluded from randomized controlled trial (*N* = 295)	Included in randomized controlled trial (*N* = 406)	*P* value
**Demographics and background**			
Age (Mean ± SD), year	65 ± 18	66 ± 17	0.411
Gender (female)	101 (34.2%)	151 (37.2%)	0.421
Country			0.000
Israel	274 (92.9%)	270 (66.5%)	
Greece	16 (5.4%)	76 (18.7%)	
Italy	5 (1.7%)	60 (14.8%)	
Admitted from home	204 (69.2%)	276 (68%)	0.742
BMI, kg/m^2^	27.1 (6.7)	27.4 (5.8)	0.610
Charlson Score (Mean ± SD)	2 ± 2	2 ± 2	0.497
Dementia	17 (5.8%)	49 (10.7%)	0.050
Diabetes	61 (20.7%)	90 (22.2%)	0.636
Chronic kidney disease	71 (24.1%)	79 (19.5%)	0.129
Hematological Malignancy	25 (8.5%)	14 (3.4%)	0.004
Congestive heart failure	66 (22.4%)	92 (22.7%)	0.928
Chronic pulmonary disease	57 (19.3%)	91 (22.4%)	0.322
Immune suppressive therapy	54 (18.3%)	61 (15%)	0.247
Known colonization by pathogen before infection	69 (23.4%)	96 (23.6%)	0.937
Recent surgery	83 (28.1%)	114 (28.1%)	0.987
**Status at infection onset (culture taken time)**			
Temperature, °C (SD)	37.9 (1.7)	38.0 (1.7)	0.655
Systolic blood pressure, mm Hg (SD)	106 (24)	109 (21)	0.054
Haemodynamic support	68 (24.2%)	75 (18.5%)	0.069
Mechanical ventilation (invasive)	198 (69.5%)	264 (65%)	0.221
Haemodialysis	11 (3.9%)	27 (6.7%)	0.118
SOFA score (Mean ± SD)	6 ± 3	6 ± 3	0.755
Creatinine Clearance (Cockcroft-Gault Equation), mL/min (Percentiles 25–75)	59.79 (32.54–108.58)	69.95 (41.21–126.27)	0.012
Albumin, g/dL (SD)	2.3 (0.6)	2.4 (0.7)	0.285
White blood cells, thousands/mL (SD)	13.22 (9.85)	14.12 (8.89)	0.212
Arterial line	76 (25.8%)	151 (37.2%)	0.001
Central venous catheter	119 (40.3%)	225 (55.4%)	0.000
Urinary catheter	228 (77.3%)	354 (87.2%)	0.001
Nasogastric tube	201 (68.1%)	285 (70.2%)	0.559

### Infection characteristics and antibiotic treatment

Severity of infection was similar in the two groups, as evidenced by similar SOFA scores, need for hemodynamic support, blood pressure and body temperature. Patients not randomized were less likely to acquire their infection in the intensive care unit [22.7% (67/295) vs. 30.5% (124/406). *p* = 0.022], to be infected with Enterobacteriacaeae [35/295 (11.9%) vs. 73/406 (18%), *p* = 0.027]; and more likely to have urinary tract infection [32/295 (10.8%) vs. 26/406 (6.4%), *p* = 0.035]. The minimum inhibitory concentration (MIC) of > 0.5 mg/L for colistin was more prevalent in randomized patients [24.3% (85/350) vs. 7.7% (18/233), *p* = 0.000] [15].

RCT patients received higher rates of colistin-meropenem combination therapy than non-randomized patients [51.2% (208/406) vs. 31.9% (53/166), *p* = 0.000]. Colistin loading dose was administered more often to randomized patients [92.6% (376/406) vs. 73.5% (122/166), *p* = 0.000]. No difference was observed in mean colistin maintenance dose per day between the two groups. Among 14-day survivors, treatment with colistin was longer in randomized patients than in non-randomized patients [13 days (IQR 10–16) vs. 8.5 days (IQR 0–15), *p* = 0.000] (Table 2).

**Table 2:** Infection characteristics and antibiotic treatment^a^

	Excluded from randomized controlled trial (*N* = 295)	Included in randomized controlled trial (*N* = 406)	*P* value
**Infection characteristics**			
Acquisition of infection in the intensive care unit	67 (22.7%)	124 (30.5%)	0.022
Pathogen			
Acinetobacter baumannii	236 (80%)	312 (76.8%)	0.318
Enterobacterales	35 (11.9%)	73 (18%)	0.027
Pseudomonas/other	24 (8.1%)	21 (5.2%)	0.114
Type of infection			
Bacteraemia	109 (36.9%)	173 (42.6%)	0.131
Ventilator-associated or hospital-acquired pneumonia	140 (47.5%)	182 (44.8%)	0.490
Probable ventilator-associated pneumonia	14 (4.7%)	25 (6.2%)	0.421
Urinary tract infection	32 (10.8%)	26 (6.4%)	0.035
Colistin MIC distribution > 0.5 mg/L	18 (7.7%), *n* = 233	85 (24.3%), *n* = 350	0.000
**Antibiotic treatment**			0.000
Colistin	113 (68.1%), *n* = 166	198 (48.8%)	
Colistin and meropenem	53 (31.9%), *n* = 166	208 (51.2%)	
Colistin loading dose	122 (73.5%), *n* = 166	376 (92.6%)	0.000
Treatment days in patients alive ≥14 days, median (Percentiles 25–75)	8.5 (0–15), *n* = 200	13 (10–16), *n* = 273	0.000
Mean colistin maintenance dose per day, million units (percentiles 25–75)			
Creatinine clearance< 50 ml/min	4.2 (2.1–6.0)	4.0 (3.0–6.0)	0.629
Creatinine clearance≥50 ml/min	8.6 (5.8–9.0)	8.5 (7.0–9.0)	0.239

^a^Numbers apply to all patients in the group unless stated otherwise

### Outcomes

More non-randomized patients met the criteria for the primary outcome, clinical failure at day 14, than randomized patients [82% (242/295) vs. 75.5% (307/406), *p* = 0.042]. Mortality rates were higher in non- randomized patients [40.2% (117/295) vs. 33% (134/406 in the RCT patients, *p* = 0.051]. The difference between the two groups waned at the end of study: 28-day mortality was 47.8% (138/295) in the non- randomized patients vs. 44.3% (180/406) in RCT patients.

Univariate analysis for clinical failure at day 14 is displayed in Table 3.

**Table 3:** Univariate analysis for clinical failure at day 14^a^

	Clinical success at day 14 (*N* = 152)	Clinical failure at day 14 (*N* = 549)	*P* value
Age (Mean ± SD), year	62.79 (18.514)	66.08 (16.975)	0.038
Gender (female)	68 (44.7%)	184 (33.5%)	0.011
Country			0.001
Israel	113 (74.3%)	431 (78.5%)	
Greece	32 (21.1%)	60 (10.9%)	
Italy	7 (4.6%)	58 (10.6%)	
Hematological malignancy	2 (1.3%)	37 (6.7%)	0.010
Congenative heart failure	25 (16.4%)	133 (24.2%)	0.042
Arterial line	35 (23%)	192 (35%)	0.005
Chronic pulmonary disease	23 (15.1%)	125 (22.8%)	0.041
Systolic blood pressure, mm Hg (SD)	111.97 (20.539)	106.66 (22.499)	0.009
Haemodynamic support	16 (10.6%)	127 (23.7%)	0.000
Mechanical ventilation (invasive)	81 (53.6%)	381 (70.6%)	0.000
Haemodialysis	1 (0.7%)	37 (6.9%)	0.003
Creatinine clearance (Cockcroft-Gault Equation), mL/min (Percentiles 25–75)	72.60 (41.16–132.14)	64.01 (36.08–118.69)	0.199
Albumin, g/dL (SD)	2.46 (0.678)	2.327 (0.6383)	0.035
Nasogastric tube	92 (60.5%)	394 (71.8%)	0.008
Pathogen			
*Acinetobacter baumannii*	100 (65.8%)	448 (81.6%)	0.000
*Enterobacteriacaeae*	35 (23%)	73 (13.3%)	0.003
*Pseudomonas*/other	17 (11.1%)	28 (5.1%)	0.007
Type of infection			
Bacteraemia	68 (44.7%)	214 (39%)	0.200
Ventilator-associated or hospital-acquired pneumonia	55 (36.2%)	267 (48.6%)	0.006
Probable ventilator-associated pneumonia	10 (6.6%)	29 (5.3%)	0.537
Urinary tract infection	19 (12.5%)	39 (7.1%)	0.033
Acquisition of infection in the intensive care unit	24 (15.6%)	167 (30.4%)	0.000
Exclusion from the RCT	53 (34.9%)	242 (44.1%)	0.042
Colistin MIC distribution > 0.5 mg/L	27 (21.3%), *n* = 127	76 (16.7%), *n* = 456	0.230
Antibiotic treatment			
Combination arm: colistin and meropenem	68 (50.7%), *n* = 134	193 (44.1%), *n* = 438	0.174
No loading dose	12 (9.0%), *n* = 134	62 (14.2%), *n* = 438	0.117
Treatment days in patients alive ≥14 days, median (Percentiles 25–75)	13 (8–16)	8 (4–14)	0.000
Mean colistin maintenance dose per day, million units (Percentiles 25–75)	7.9 (5.0–9.0)	7.2 (4.0–9.0)	0.330

^a^Numbers apply to all patients in the group unless stated otherwise

At multivariable logistic regression, male gender, age, hemodynamic support, and acquisition of the infection in the intensive care unit were associated with higher rates of 14-day clinical failure. *Pseudomonas*/other bacteria as initial isolate were associated with lower rates of 14-day clinical failure. Non-inclusion in the RCT was not an independent risk factor for clinical failure at day 14 (Table 4).

**Table 4:** Logistic regression analysis of independent risk factors for clinical failure at day 14 of infection onset

	OR (95% CI)	*P* value
Exclusion from the RCT	1.341 (0.818–2.200)	0.245
Age^a^	1.018 (1.005–1.031)	0.006
Gender (female)	0.543 (0.345–0.854)	0.008
*Enterobacterales*	0.658 (0.361–1.202)	0.173
*Pseudomonas*/other	0.416 (0.183–0.416)	0.037
Systolic blood pressure, mm Hg^b^	0.992 (0.981–1.002)	0.119
Haemodynamic support	2.561 (1.188–5.520)	0.016
Mechanical ventilation (invasive)	1.481 (0.920–2.384)	0.106
Acquisition of infection in the intensive care unit	2.061 (1.170–3.632)	0.012

*N* = 701Akaike’s information criterion goodness of fit = 516.012; constant: β = 2.311; risk for clinical failure at day 14: OR > 1

^a^Age- per 1 year increment

^b^Systolic blood pressure - per 1 mm Hg increment

## Discussion

In our study, patients not randomized in the trial were similar to randomized patients in their baseline characteristics, though RCT patients showed minor differences towards a more severe infection. They had more lines and catheters and acquired their infection more often in the intensive care unit. Non- randomized patients were less infected by *Enterobacteriaceae*, showed lower MIC distributions for colistin, and were presented with higher rates of urinary tract infection.

Univariate analysis showed that non- randomized patients were more inclined to clinical failure on day 14 from infection onset. However, on multivariate analysis exclusion from the RCT was not an independent risk factor for clinical failure.

The major reason for exclusion from the RCT was refusal of the patient, the legal guardian, or the treating physician to participate in the trial. In this study, we were authorized by the local ethics committees to recruit patients who were not able to provide informed consent and did not have a legal guardian, with the consent of an approved independent physician or a family member (as described in the Ethics section). This allowed the inclusion of severely ill patients that characterize the AIDA trial. On the other hand, patient refusal implied that patients who were able to consent refused randomization, and this translated into the inclusion of less severely ill patients in the observational arm. Non-randomized patients suffered more often from hematological malignancies. This could be a result of the patients’ or treating physicians’ concern regarding the inclusion of a patient with a compromised immune system. Creatinine clearance levels were lower in non- randomized patients, perhaps reflecting the reluctance to include patients with impaired kidney function into a trial involving a nephrotoxic drug such as colistin.

No significant difference between colistin monotherapy and combination therapy was observed for clinical failure at day 14 in included and excluded patients. Per AIDA RCT protocol, ~ 50% of patients received colistin-meropenem combination therapy. Colistin was administered as a 9-million-unit (MIU) loading dose followed by maintenance doses, with a minimum treatment period of 7 days. Non-randomized patients received mainly colistin monotherapy, reflecting the standard of care, with a lower rate of colistin loading dose administration and a shorter treatment period. The difference in management and the significantly related variates described in the logistic regression can explain the higher rates of clinical failure in non- randomized patients.

In a trial published in 2015, Paul et al. examined the external validity of a RCT comparing trimethoprim-sulfamethoxazole versus vancomycin for the treatment of invasive methicillin-resistant *Staphylococcus aureus* (MRSA) infections. The major point of difference from AIDA study was that patients that were not able to sign an informed consent and did not have a legal guardian could not enter the MRSA RCT- thus excluded patients were more ill than included patients, and the differences between the two populations were more substantial, including primary outcomes, with excluded patients showing significantly higher clinical failure and 30-day all-cause mortality rates [5].

In order to minimize differences between the study sample and “real-world” patients, the AIDA RCT did not exclude patients for underlying conditions or sepsis severity while taking into account the potential compromise of internal validity caused by increasing heterogeneity of the recruited patients. This is of major importance, especially in comparison with registration or pharmaceutical company-sponsored trials. Ha et al. examined the proportion of patients encountered during routine clinical practice who would qualify for enrollment into a pivotal RCT of biological agents for inflammatory bowel disease (IBD). In this retrospective cohort study, the eligible patients were examined for inclusion in at least one of seven selected published RCTs. Only ~ 30% of patients would have qualified for enrollment due to numerous exclusion criteria [16]. A literature review published in 2015 identified the use of restrictive inclusion/exclusion criteria as one of the key factors that limited external validity of trial findings [17]. This issue raises the importance of designing an RCT to include a diverse population with limited exclusion criteria so that the results can be generalized to the population in hand.

Our study has few limitations. First, the observational cohort included patients excluded due to three out of seven exclusion criteria which account for most of the observational sample [81.7% (295/361)], thus not all RCT excluded patients entered the observational arm. We chose to focus on these exclusion criteria since they truly reflect patients compatible for recruitment. Second, this study focuses on one aspect of external validity- comparison of characteristics and outcomes of excluded and included patients. This aspect refers to the population validity component and addresses the question of whether the findings of a study can be generalized to patients with characteristics that are different from those in the study, or patients who are treated or followed up differently. For a broader evaluation of external validity, it will be interesting to test ecological validity which specifically examines whether the findings of a study can be generalized to different clinical settings in everyday life.

## Conclusions

The similarity between patients in the observational and RCT arms has strengthened our confidence in the population external validity of the AIDA trial. Limited exclusion criteria and access to recruiting the most severely ill patients into the trial population are key elements conferring the high population external validity in the AIDA trial, and overall for this type of infectious disease trials. Extending the RCT to include an observational study arm strengthens and optimizes the evidence emerging from the study. The other major benefit of a hybrid study is that it alleviates concerns in real life clinical implementation.

## Data Availability

The datasets used and/or analysed during the current study are available from the corresponding author on reasonable request.

## Abbreviations

RCT: Randomized controlled trial
CR GNB: Carbapenem-resistant gram-negative bacteria
SOFA: Sequential organ failure assessment
MIC: Minimum inhibitory concentration
IBD: Inflammatory bowel disease
MIU: Million units
